# The effect of ball pressure and maximal isometric neck strength on head acceleration during purposeful heading in adult football players during heading drills

**DOI:** 10.1016/j.jsampl.2023.100051

**Published:** 2023-12-16

**Authors:** Ethan Pereira, Kerry Peek, Chad McLean, Andrew P. Lavender, Fadi Ma'ay, Paul Davey, Susan Morris, Julia Georgieva

**Affiliations:** a Curtin School of Allied Health, Curtin University, Perth, WA, 6102 Australia; b Sydney School of Health Sciences, The University of Sydney, Camperdown, NSW, 2006 Australia; c Institute of Health and Wellbeing, Federation University Australia, Ballarat, VIC, 3350 Australia

**Keywords:** Soccer, Head impact, Cervical muscle strength

## Abstract

**Objective:**

This randomised repeated measures study explored the effect of ball pressure and maximal isometric neck strength on head acceleration during purposeful heading in adult football players during heading drills within a laboratory environment.

**Methods:**

Recreational football players (n ​= ​17) attended one familiarisation session to determine baseline maximal isometric neck strength, followed by two experimental sessions where they randomly trialled two conditions (>72-h apart). The first condition included 20 rotational headers with a match-ball at low-pressure (58.6 ​kPa; 8.5 psi) and the second included 20 rotational headers with a match-ball at high-pressure (103.4 ​kPa; 15.0 psi) whilst instrumented with an inertial measurement unit.

**Results:**

A statistically significant difference between conditions for both peak linear head acceleration (F ​= ​15.2; p= ​ < ​ 0.001) and peak angular head velocity (F ​= ​5.71; p ​= ​0.018) during purposeful heading. The low-pressure ball condition demonstrated a 12 ​% reduction in peak linear acceleration and 6 ​% reduction in peak angular velocity when compared with high-pressure ball condition. Additionally, neck strength significantly predicted head acceleration during purposeful heading (p ​ = ​ <0.05).

**Conclusion:**

These findings suggest that lower ball pressure and higher neck strength can lower head acceleration during heading in adult football players during heading drills within a laboratory environment.

## 1 Introduction

Unique to football (soccer) is a skill known as purposeful heading, whereby players intentionally use their unprotected heads to strike the ball [1,2]. Ball-to-head impact result in linear and angular acceleration of the skull, with purposeful heading being found to induce short-term microstructural and functional changes in the brain [2,[3], [4], [5]]. Rotational headers, whereby a player redirects the ball perpendicularly with their head, are of particular interest due to their common use in training sessions and match situations [2]. These types of headers have been reported to cause short-term cognitive and electrophysiological impairments, which typically normalised within 24 ​h post-heading [2]. Despite evidence of short-term changes in the brain [2], the cumulative and long-term consequences of repetitive head impacts from purposeful heading are not yet known [1,6]. Given that football players routinely practise purposeful headers during training and may head the ball 6–12 times per competitive match [3], taking a proactive approach to identify strategies that will aid in reducing the impact of repeated head acceleration to protect the long-term brain health of football players appears justified.

One component affecting head acceleration during heading is ball pressure. Current International Football Association Board (IFAB) guidelines state that footballs used during competitive matches should have a pressure range between 58.6 and 107.6 ​kiloPascal (kPa) (or 8.5 to 15.6 pounds per square inch (psi) [7,8]. Ball pressure has been shown to influence the recoil force magnitude of the head during ball-to-head impacts [7,8]. Computational models have demonstrated that balls with lower ball pressure lead to lower head acceleration during purposeful heading with emerging evidence in human participants [1,9].

Additionally, neck strength is another component believed to influence head acceleration during heading [1], whereby, football players activate their neck musculature to increase their effective mass in anticipation of a head-to-ball contact [1,3,9]. A systematic review [1] reported a relationship between higher peak isometric neck muscle strength and lower head acceleration during purposeful heading. Higher neck muscle strength is purported to reduce head acceleration during heading by controlling head movement and increasing head-trunk stabilisation to prevent their head from recoiling backwards or sideways during ball to head impacts [3,9,10].

Considering that both ball pressure and neck strength are modifiable risk factors, it is important to further investigate what effect they have on head acceleration during purposeful heading, as these data could be used to inform future heading guidelines or practices.

The primary objective of this study is to determine the effect of ball pressure on head kinematics (peak linear acceleration and peak angular velocity) during purposeful heading in adult football players during simulated heading drills within a laboratory environment. The secondary objective is to determine the effect of maximal isometric neck strength on head acceleration during purposeful heading of low- and high-pressure match balls in adult football players.

Our hypotheses are that lower ball pressures will result in lower head accelerations during heading; and players with higher maximal isometric neck strength will demonstrate lower head accelerations during heading.

## 2 Methods

### 2.1 Study design

A randomised repeated measures design, with at least 72 ​h between tests, was used to ascertain the effect of two separate conditions: i) 20 rotational headers with low-pressure match ball (58.6 ​kPa; 8.5 psi); and ii) 20 rotational headers with high-pressure match ball (103.4 ​kPa; 15.0 psi). Prior to attending the experimental sessions, each participant attended a familiarisation session to gather baseline neck measures and to get acquainted with the heading protocol.

### 2.2 Recruitment

Nineteen healthy recreational adult football players aged 18–35 years were recruited to participate in this study. Participants were included in the study if they had played football for at least one year and were confident and comfortable with purposeful heading. Participants were excluded if they presented with any history of the following: i) head or neck injury within the past 12 months; ii) diagnosed concussion within the past 12 months; iii) any brain injury resulting in a loss of consciousness; iv) any diagnosed neurological condition [2]. Participants were also excluded if they played exclusively as a goalkeeper, as players in this position do not routinely head the ball [2]. This study was approved by Curtin University Human Research Ethics Committee (HRE2021-0644) and informed consent was obtained from all participants prior to data collection.

### 2.3 Experimental procedures

#### 2.3.1 Familiarisation session

At the familiarisation session, anthropometric data (body mass, height and neck girth) and demographic data (age, sex and playing experience) were recorded. Maximal voluntary isometric neck muscle strength was measured using an isokinetic dynamometer (Humac Norm, Computer Sports Medicine, Inc. MA, USA). Participants were tested in a seated position, with their head in neutral and their seventh cervical vertebra in line with the axis of rotation of the torque arm [11,12]. Resistance pads were placed on the participant's forehead and occipital protuberance. A Velcro strap was wrapped around the participant's head to secure it to the dynamometer arm [12]. Participants were instructed to exert a maximal force against the static resistance pad for 5 ​s. Each participant was given one practise trial and two recorded trials in the direction of both flexion and extension, with a 45 ​s rest in between each effort. Of the two recorded trials, the peak values for isometric flexor and peak isometric extensor strength values were chosen for data analysis and normalised to body mass. Following neck strength measures, participants were given an opportunity to practise the heading protocol to minimise later learning effects. Participants were given at least 72 ​h prior to taking part in the experimental session to allow for an adequate rest of neck muscles [11,12].

#### 2.3.2 Experimental sessions

We chose to use heading trials within a laboratory environment to control and standardise the conditions as much as possible. To further ensure inter-sessional standardisation, participants were instructed to abstain from vigorous physical activity and heading for 24 ​h prior to attending the experimental session. An inertial measuring unit (IMU) (Blue Trident, Vicon Motion Systems Ltd, Oxford, UK) was used to measure linear acceleration (tri-axial accelerometer) and angular velocity (tri-axial gyroscope) of the head during each header based on a previously published protocol [8]. This device was placed centrally at the base of the occiput and secured using a close-fitting elastic ‘swim cap’ [8]. For the heading protocol, a standard size 5 match ball (Deploy Football, Taren Point, NSW, Australia) was used, with only the ball pressure varying depending on the predetermined condition for each participant. Each football was projected at a velocity of 30 kph using a football delivery device (Ball Launcher, Globaltec Innovation LTD, Brightlingsea, Essex, UK) positioned 6-m from the participant [2]. This ball velocity was measured using a Stalker Sport 2 radar gun (Stalker Sport, Richardson, Texas, USA) was chosen to represent a common game scenario in which the ball is delivered at a lower velocity, rather than from a goal kick where ball velocities might reach as high as 85 kph [13]. Each ball was delivered at head height on a downward arc to simulate a free play header typically observed during training session or football match [2]. Participants were instructed to keep their feet on the ground and to redirect the football perpendicularly to its initial trajectory [2,8], aiming for a small goal, with a height of 1 ​m and a width of 2 ​m, 90° to their left. Participants performed 20 rotational headers over a period of 10 ​min [2]. For a header to be valid, participants needed to keep their feet grounded [9]. To reduce the cumulative burden of head impacts, invalid headers were only repeated if the participant did not make head contact with the ball [8]. Following the experiment, participants were asked to abstain from vigorous physical activity for the remainder of the day. After a rest of at least 72 ​h, participants returned to complete testing in the remaining condition.

### 2.4 Sample size

An *a priori* minimum sample of size of 16 participants was determined to show an effect size of f ​= ​0.33 (equivalent to partial eta squared ​= ​0.1 or a 10 ​% difference in outcome between ball pressures) with 80 ​% power using a within factor repeated measures ANOVA.

### 2.5 Data analysis

Following each experimental session, accelerometer and gyroscope data recorded by the IMU were exported into an Excel® spreadsheet. Peak linear acceleration and peak angular velocity were extracted for each header. SPSS (SPSS 17, Chicago, IL, USA) was used for statistical analysis. A linear mixed effects models was used to determine differences in peak linear acceleration and angular velocity between conditions (low-pressure ball and high-pressure ball). An independent t-test was conducted to determine if there was a significant difference in peak linear acceleration or peak angular velocity based on ball pressure. Finally, separate direct-entry multiple regression analyses were performed for peak isometric neck flexor and peak isometric neck extensor strength to determine which variables predicted peak linear acceleration and peak angular velocity. Significance was set *a priori* at α ​= ​0.05.

## 3 Results

Of the 19 participants recruited to participate in the study, two participants were excluded due to a recent diagnosed concussion. The final cohort included 17 participants (females n ​= ​3; mean ​± ​SD; age 22 ​± ​3.5 years; height 177.5 ​± ​7.9 ​cm; mass 76.2 ​± ​14 ​kg; neck girth 37.5 ​± ​3.5) with playing experience ranging from 2 to 20 years (mean ​± ​SD 10.1 ​± ​5.9 years). Table 1 summarises peak linear acceleration and peak angular velocity for male and female players during heading.

**Table 1:** Summary of peak linear acceleration and peak angular velocity data for male and female players during purposeful heading.

	Peak linear acceleration (g)				Peak angular velocity (rad/s)			
	Min	Max	Mean (95 ​% CI)	Median	Min	Max	Mean (95 ​% CI)	Median
Male players	4.2	28	13 (12.5–13.5)	12	2.7	47.6	14.9 (14.4–15.4)	13.4
Female players	7.1	56.2	15 (14–15.8)	13.7	7	45.4	16.2 (14.6–17)	14.4

### 3.1 Effect of ball pressure on head acceleration during purposeful heading

The result of the linear mixed effect model demonstrated a statistically significant difference between ball pressure and both peak linear acceleration (F ​= ​15.2; p ​ = ​<0.001) and peak angular velocity (F ​= ​5.71; p ​= ​0.018) of the head during purposeful heading during simulated heading drills within a laboratory environment. Table 2 summarises the data for peak linear acceleration and peak angular velocity of the head for each ball pressure. An independent T-test revealed a statistically significant difference in peak linear acceleration and peak angular velocity of the head between low- and high-pressure balls (p ​ = ​ <0.05). An example of the time series data for one participant can be found in Appendix I.

**Table 2:** Summary of peak linear acceleration and peak angular velocity of the head for each ball pressure.

	Peak linear acceleration (g)				Peak angular velocity (rad/s)			
Ball pressure (kPa)	Min	Max	Mean (95 ​% CI)	Median	Min	Max	Mean (95 ​% CI)	Median
Low pressure (58.6)	4.2	33.4	12.8 (12–13.3)	11.8	2.7	43.8	15.3 (14.1–15.4)	14.2
High pressure (103.4)	7.1	56.2	14.4 (13.7–14.7)	13.4	7	47.6	16.2 (14.5–16)	15

### 3.2 Effect of neck strength on head acceleration during purposeful heading

For peak linear head acceleration, 17 ​% of the variance was explained by peak isometric neck flexor strength and 19 ​% of the variance was explained by peak isometric neck extensor strength. For peak angular velocity of the head, 11 ​% of the variance was explained by peak isometric neck flexor strength and 7 ​% by peak isometric neck extensor strength. Table 3 summarises the strength related predictors (regression analyses) for peak linear acceleration and peak angular velocity of the head during purposeful heading during simulated heading trials.

**Table 3:** Summary of strength related predictors for linear acceleration and angular velocity.

	Linear acceleration (g)			Angular velocity (rad/s)		
Variable	R^2^	p-value	F	R^2^	p-value	F
Peak isometric neck flexor strength	0.17	0.031∗	15.6	0.11	<0.001∗	8.4
Peak isometric neck extensor strength	0.19	<0.001∗	18.5	0.07	0.021∗	6.48

Key: ∗ Statistically significant

## 4 Discussion

This study demonstrated that head kinematics, (peak linear acceleration and peak angular velocity), are influenced by ball pressure and maximal isometric neck strength during purposeful heading in adult football players during heading trials within a laboratory environment. These findings support the study's hypotheses that head acceleration during heading is lower when heading balls with lower ball pressures, and if players have higher maximal isometric neck strength.

### 4.1 Ball pressure

Our results show a statistically significant relationship between lower ball pressure and lower head acceleration during purposeful heading in adult football players during heading trials. We observed a 12 ​% reduction in peak linear acceleration and a 6 ​% reduction in peak angular velocity when heading with a low-pressure ball (58.6 ​kPa) compared with a high-pressure ball (103.4 ​kPa). The results from this study are consistent with mathematical models that predict lower inflation pressure reduces head acceleration during head-to-ball impacts [9,14]. Two previous studies have evaluated the relationship between ball pressure and head acceleration using human participants [8,15]. Consistent with our results, these studies also reported significant reductions in head acceleration with reduced ball pressure [8,15]. However, the lowest inflation pressures used within these studies were below the IFAB regulated range [8,15]. To the best of the authors knowledge, our study is the first to compare head acceleration during purposeful heading using match balls inflated close to the highest and lowest ball inflation pressures recommended by IFAB. Our study provides evidence that significant reduction in peak linear acceleration and peak angular velocity of the head is attainable by reducing ball pressure from 103.4 ​kPa (15 psi) to 58.6 ​kPa (8.5 psi) (from near the maximum to minimum end of IFAB ball regulations). Using a head form model, Auger et al. [16] reported that the same reduction in ball pressure yields a 20 ​% reduction ball impact force to the head. It is suggested that lower ball pressure increases the contact time between the ball and the head, consequently reducing the recoil force magnitude experienced by the head during heading [7,9,15]. Whilst our study supports previous literature that head acceleration during heading can be reduced by using lower ball pressure, it is uncertain how this will affect football game play [14], particularly as lowering inflation pressure reduces the speed of the ball [9]. Although slower balls may reduce shot speed, the benefit could be greater player control and touch [9,14]. This will provide players with greater passing and shooting accuracy which may lead to an increase in goal scoring opportunities [9]. As a result, it's possible that using lower pressure balls may be beneficial not only from a long-term injury risk perspective by potentially reducing the accumulative acceleration load that a player will experience over the course of their career but may also have game performance benefits as well [9]. Ensuring footballs are on the lower end of the IFAB permitted ball pressure range is an easily implementable strategy using a pressure gauge to measure ball pressure before training and games.

### 4.2 Neck strength

Our findings demonstrate that both peak isometric neck flexor and peak isometric neck extensor strength significantly predicted peak angular velocity and peak linear acceleration of the head during heading in adult football players during heading trials. Previous studies have also reported that higher neck strength is associated with reduced head acceleration during purposeful heading [17,18,19,20]. Further, Tierney et al. [21] reported that female football players demonstrated statistically significant lower neck strength and higher head accelerations during heading in comparison to male football players. Due to the limited number of female participants in our study, we cannot draw the same conclusions. However, it is suggested that football players with stronger neck muscles have greater tensile stiffness and a greater cross-sectional area [1]. This in turn increases the effective mass of the player, which refers to the mass of the player that can oppose the force of the ball during head to ball impacts [1,9]. With strength being a modifiable predictor of head acceleration, further research is recommended on the effect of neck exercises on head kinematics during heading for players at grassroots and professional levels. This may be especially beneficial for youth or female football players who appear to have reduced neck strength compared with male football players and who may be at greater risk of higher head accelerative loads during heading [8,21]. While there is emerging evidence that neuromuscular neck exercises can reduce head acceleration and acute injury risk associated with heading in adolescent male and female football players, there are limited data in adult players or players of different skill levels [22,23]. While the results of this study indicate that higher neck strength is associated with lower head acceleration during simulated heading trials, the contribution of heading technique requires further exploration. Heading technique, involving factors such as body positioning, timing, and the point of contact with the ball, has been reported to effectively reduce head acceleration during heading [24]. Future studies in this field should focus on further investigating the nuances of heading technique and its impact on reducing head acceleration during heading [24].

### 4.3 Strengths and limitations

A strength of our study was that the ball pressures tested were within the allowable range stipulated by IFAB, thereby providing insight into what effect the lowest and highest pressure values have on head acceleration during heading. Additionally, clear standardisation between trials was achieved by adhering to the heading protocol (supported by the use of a ball launcher to ensure uniformity in ball speed) as well as using the same participants for all trials via a randomised repeated measures design.

It is acknowledged that as headers were conducted in a controlled laboratory setting, where participants were instructed to redirect the football perpendicularly whilst standing, that these results may not translate to all types of headers exhibited during match play particularly at different ball delivery velocities. During on-field scenarios, players may head balls with different velocities, jump for aerial contests or exert maximal force with their head against the ball in an effort to score a goal. Another limitation was that a head mounted sensor was used to measure head acceleration [8,22]. which may potentially under- or over-estimate head acceleration when compared with mouthguard sensors [22,25]. It is also acknowledged that the head-mounted IMU gives an indication of head acceleration around the IMU-location point rather than the centre of gravity of the head (which we did not correct for). However, the decision was made deliberately *a priori* to use a head-mounted sensor in favour of an instrumented mouthguard in an attempt to reduce the potential confounding effect of teeth clenching on neck muscle recruitment during the heading trials, given that our participants were unused to wearing mouthguards (which may also have changed heading technique making the data less ecologically valid). We followed a protocol for the use of head mounted sensors as described in an earlier heading study with each participant and every heading trial being subject to the same conditions [8]. Given these limitations we must advise caution when comparing these data to data collected in other studies using different measurement devices and mounting locations. Additionally, the majority of our participants were male with insufficient number of female participants to enable a sub-group analysis by sex. However, it is noted that male players demonstrated a mean peak linear acceleration of 13 ​g and mean peak angular velocity of 14.9 ​rad/s compared with female players who demonstrated higher mean values for both peak linear acceleration (15 ​g) and peak angular velocity (16.2 ​rad/s). These results are within the lower end of the ranges reported in an earlier review paper on heading [24] which also reported that in studies where head kinematics was directly compared between male and female players, females players demonstrate higher values [24]. However, making direct comparisons of head kinematic values between studies is challenging due to a range of confounders which can influence the results (including age, sex and skill level of players, type of IMU used and the placement of the IMU, ball speed, type of header and so forth).

Finally, it must be acknowledged that our chosen outcome measure of head kinematics may not correspond to acute or long-term injury risk related to brain tissue stress or strain during heading [24,26]. While earlier studies have reported that injury risk depends on many factors, head impact magnitude being one of them [26,27], the direct effect of reduced head acceleration on brain injury risk needs further exploration.

## 5 Conclusion

This study provides evidence that lower ball pressure and higher neck strength can reduce head kinematics (peak linear acceleration and peak angular velocity) during purposeful heading in adult football players during heading trials within a controlled laboratory setting. Further research is needed to explore whether similar effects of ball pressure on head acceleration is observed during in-game scenarios as well as the effect this has on short and long-term brain injury risk.

## Confirmation of ethics

This study was approved by the Curtin University Human Research Ethics Committee (HRE2021-0644) and informed consent was obtained from all participants prior to data collection.

## Funding

None to declare.

## Data disclosure statement

Data are available from the author team on reasonable request.

## Declaration of competing interest

KP is a member of UEFA's Expert Group on Heading and Football Australia's Expert Working Group on Heading and Concussion. KP and JG are currently contracted as Injury Spotters for FIFA organised tournaments. All other authors declare no conflicts of interest.

## Figures and Tables

**Figure 1:**
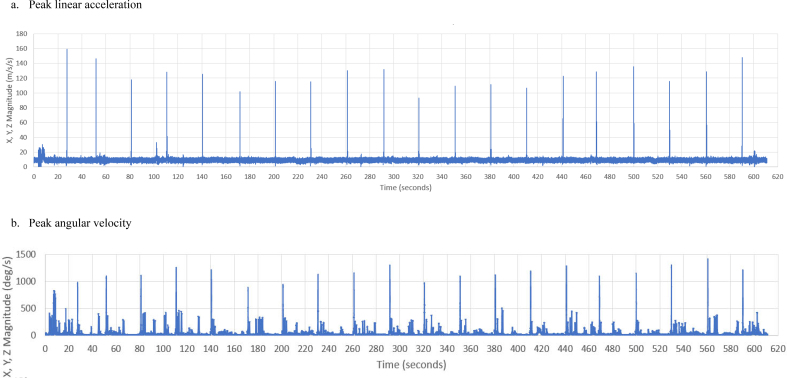
Example of IMU data from one participant showing a sample headers with the low pressure ball for peak linear acceleration and peak angular velocity.
